# Mutant USA strain of porcine circovirus type 2 (mPCV2) exhibits similar virulence to the classical PCV2a and PCV2b strains in caesarean-derived, colostrum-deprived pigs

**DOI:** 10.1099/vir.0.066423-0

**Published:** 2014-11

**Authors:** Tanja Opriessnig, Chao-Ting Xiao, Priscilla F. Gerber, Patrick G. Halbur, Shannon R. Matzinger, Xiang-Jin Meng

**Affiliations:** 1 The Roslin Institute and The Royal (Dick) School of Veterinary Studies, University of Edinburgh, Midlothian, UK; 2 Department of Veterinary Diagnostic and Production Animal Medicine, College of Veterinary Medicine, Iowa State University, Ames, Iowa, USA; 3 Department of Biomedical Sciences and Pathobiology, Center for Molecular Medicine and Infectious Diseases, College of Veterinary Medicine, Virginia Polytechnic Institute and State University, Blacksburg, Virginia, USA

## Abstract

In 2012, a mutant porcine circovirus type 2 (mPCV2) strain was identified in cases of PCV-associated disease (PCVAD) in the USA. The mPCV2 had an additional amino acid, lysine (K), in the capsid at position 234. The objectives of this study were to compare the pathogenicity of mPCV2, PCV2a and PCV2b in pigs using biologically pure infectious virus stocks derived from respective infectious DNA clones, and to investigate the importance of genotype-specific ORF2 and the presence of lysine at position 234 of the capsid. A total of 47, 2-week-old, caesarean-derived, colostrum-deprived (CDCD) pigs were assigned to one of seven groups. At 3 weeks of age, the pigs were experimentally inoculated with saline, PCV2a, PCV2b, mPCV2, PCV2b-234-K (lysine addition in ORF2), chimeric PCV2b-ORF1/mPCV2-ORF2 or reciprocal chimeric mPCV2-ORF1/PCV2b-ORF2. All pigs were necropsied 21 days post-infection (p.i.). Gross lesions were limited to visible icterus and loss of body condition in a portion of the mPCV2 pigs. The amount of PCV2 DNA was significantly higher in pigs inoculated with mPCV2 compared with PCV2b in sera at 7 days p.i. and faecal swabs at 14 days p.i. Based on lymphoid lesions, a higher prevalence of PCVAD was seen in pigs infected with PCV2s containing the additional 234-K (64.3 %) compared with those infected with a PCV2 with the regular 233 bp ORF2 (40 %). Results indicated that all PCV2 isolates were capable of inducing severe lesions and disease in the CDCD pig model, and there was no significant difference in virulence.

## Introduction

Porcine circovirus type 2 (PCV2) has been associated with a number of diseases in growing pigs, including systemic disease, respiratory disease, enteric disease, and porcine dermatitis and nephropathy syndrome, collectively known as PCV-associated disease (PCVAD) (Opriessnig *et al.*, 2007). PCV2 is a small, single-stranded, non-enveloped, circular DNA virus containing a genome of 1766–1768 nt (Cheung *et al.*, 2007; Tischer *et al.*, 1974). There are two main ORFs: ORF1 encoding two proteins associated with virus replication designated Rep and Rep′, and ORF2 encoding the capsid protein (Finsterbusch & Mankertz, 2009; Nawagitgul *et al.*, 2000).

Several PCV2 genotypes have been recognized and designated with consecutive lower-case letters, i.e. PCV2a, PCV2b, PCV2c, PCV2d and PCV2e (Dupont *et al.*, 2008; Gagnon *et al.*, 2007; Guo *et al.*, 2010; Jantafong *et al.*, 2011). There is still an ongoing debate on the nomenclature of PCV2 genotypes; however, it is generally accepted to use the guidelines established by the European Union consortium on PCV diseases (http://www.pcvd.net) which recommends that the ORF2 sequences of PCV2 are assigned to different genotypes when the genetic distance between them is at least 0.035 (Grau-Roma *et al.*, 2008). Amongst all PCV2 genotypes, PCV2a was the predominant strain prior to 2000 and PCV2b is currently the main genotype in the global pig population (Segalés *et al.*, 2008). Interestingly, initial introduction of PCV2b in PCV2a-infected herds was commonly associated with severe PCVAD outbreaks (Cheung *et al.*, 2007; Gagnon *et al.*, 2007). However, evidence from experimental inoculations suggested that PCV2a and PCV2b were similar in virulence (Fort *et al.*, 2008; Opriessnig *et al.*, 2008), although differences in virulence have been documented within subtypes (Opriessnig *et al.*, 2006b).

In the USA, several epidemiological investigations of PCV2 genotypes have been conducted (Fenaux *et al.*, 2000; Shen *et al.*, 2010a, 2012), and PCV2a and PCV2b were the only genotypes identified. In 2012, a variant PCV2 strain with an elongation of ORF2 by one amino acid, lysine (K), was detected in several PCVAD cases (Xiao *et al.*, 2012). This strain, which is also known as mPCV2, has a shift from TTA to CTT in the genomic sequence resulting in a mutation of the stop codon (from UAA to AAG) in the ORF2, subsequently leading to expression of the additional amino acid residue lysine (Guo *et al.*, 2011). An almost identical mPCV2 had been identified previously in China (Guo *et al.*, 2010); however, sequences deposited in the GenBank database indicated that the mPCV2 virus was already present in China by 2006 (GenBank accession numbers JX679498 and JQ653449) and mPCV2 variants can be traced back to 2002 in China (Wang *et al.*, 2009). When compared with the classical Chinese PCV2a and PCV2b isolates using a Chinese conventional pig model, enhanced virulence of mPCV2 was suggested (Guo *et al.*, 2012).

The objectives of this present study were to construct an infectious clone of the USA mPCV2 in order to prepare a biologically pure infectious stock of mPCV2, to compare the pathogenicity of mPCV2, PCV2a and PCV2b side-by-side in a caesarean-derived, colostrum-deprived (CDCD) pig model, and to further investigate the importance of PCV2-genotype-specific ORF2 and the addition of the amino acid lysine at position 234 by utilizing chimeric and reciprocal chimeric PCV2b/mPCV2 viruses.

## Results

### Confirmation of the correct virus genotype in each group

Differential PCR on serum samples collected at 21 days post-infection (p.i.) indicated the presence of the correct ORF2 sequences in each of the groups except for the chimeric PCV2 group where the ORF2 of both PCV2b and mPCV2 was detected. Further sequencing analysis confirmed the results suggestive of a contamination. Therefore, the results for this particular group were excluded and not analysed. However, excluding this group from the analysis had no affect on the data interpretation or conclusion of this study, as the remaining groups of animals in this large animal study generated sufficient data to draw a conclusion.

### Clinical observation and mean daily weight gain

Signs of illness were not recognized in the pigs in the 21 day duration of the study except for the last day where visible icterus and loss of body condition were observed in three of eight mPCV2 pigs. The mean daily weight gain was not different between groups (data not shown).

### Anti-PCV2 antibody levels

All pigs were negative for anti-PCV2 IgG antibodies at inoculation and the majority of the pigs remained negative throughout the study due to the short 21 day duration of the study. One of eight mPCV2 pigs did seroconvert to PCV2 by 21 days p.i. and three of seven PCV2a pigs in addition to one of seven PCV2b pigs had sample-to-positive ratios within 0.1–0.2 and were considered suspect positive (data not shown).

### Prevalence and amount of PCV2 DNA in serum, nasal swabs and faecal swabs

**Fig. 1.**
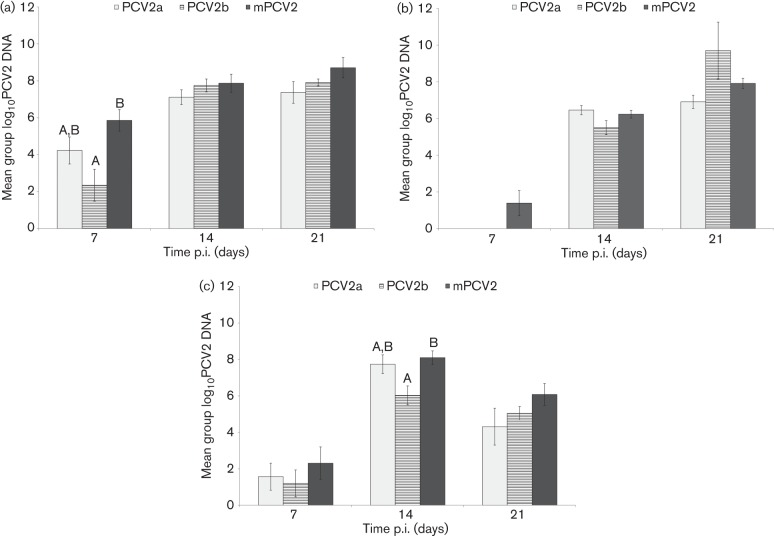
Mean group log_10_ amounts of PCV2 DNA detected in pigs at 7, 14 and 21 days p.i. with PCV2a, PCV2b or mPCV2: (a) serum samples, (b) nasal swabs and (c) faecal swabs. Different superscripts at a certain day (A, B) indicate significant differences between groups.

PCV2 DNA was not detected in any serum sample, nasal swab or faecal swab collected from the negative control pigs. The group means for PCV2a-, PCV2b- and mPCV2-infected pigs are summarized in Fig. 1, and prevalence rates and group means for all PCV2-infected groups are summarized in Table 1. In serum samples at 7 days p.i., mPCV2-infected pigs had significantly higher amounts of PCV2 DNA compared with PCV2b-infected pigs (Fig. 1a, Table 1). Moreover, the prevalence of PCV2 DNA-positive pigs was six of seven for PCV2a, four of seven for PCV2b and eight of eight for mPCV2 at 7 days p.i. Thereafter, all pigs in all infected groups were viraemic, and there was no difference amongst PCV2a, PCV2b and mPCV2 groups; however, the reciprocal chimeric PCV2 group had significantly lower levels of PCV2 DNA compared with mPCV2b by 21 days p.i. (Table 1).

**Table 1.** Prevalence and group mean±sem log_10_PCV2 genomic copies in serum samples, nasal swabs and faecal swabs collected at 7, 14 and 21 days p.i.

Group	Serum samples			Nasal swabs			Faecal swabs		
	7 days p.i.	14 days p.i.	21 days p.i.	7 days p.i.	14 days p.i.	21 days p.i.	7 days p.i.	14 days p.i.	21 days p.i.
PCV2a	6/7 (4.2±0.7)^A,B^	7/7 (7.1±0.4)	7/7 (7.4±0.6)^A,B^	0/7 (0.0±0.0)	7/7 (6.5±0.2)^A^	7/7 (6.9±0.4)^A,B^	2/7 (1.1±0.7)^A^	7/7 (7.7±0.5)^A^	6/7 (4.3±1.0)
PCV2b	4/7 (2.4±0.9)^A^	7/7 (7.7±0.4)	7/7 (7.9±0.2)^A,B^	0/7 (0.0±0.0)	7/7 (5.5±0.4)^A,B^	7/7 (9.7±1.5)^A^	3/7 (1.6±0.7)^A,B^	7/7 (6.0±0.5)^A,B^	7/7 (5.0±0.4)
mPCV2	8/8 (5.9±0.6)^B^	8/8 (7.9±0.5)	8/8 (8.7±0.5)^A^	3/8 (1.4±1.4)	8/8 (6.2±0.2)^A,B^	8/8 (7.9±0.3)^A,B^	4/8 (2.3±0.9)^A,B^	8/8 (8.1±0.4)^A^	8/8 (6.1±0.6)
PCV2b-234-K	4/6 (3.1±1.0)^A,B^	6/6 (7.5±0.2)	6/6 (8.0±0.2)^A,B^	1/6 (0.6±0.6)	6/6 (6.5±0.3)^A^	6/6 (7.4±0.3)^A,B^	6/6 (4.4±0.2)^B^	6/6 (8.2±0.4)^A^	6/6 (6.5±0.4)
Reciprocal chimeric PCV2	6/6 (4.3±0.2)^A,B^	6.7±0.4	6/6 (6.7±0.5)^B^	3/6 (2.2±1.0)	6/6 (5.0±0.4)^B^	6/6 (5.9±0.5)^B^	6/6 (4.1±0.3)^B^	5/6 (5.1±1.0)^B^	5/6 (4.0±0.8)

Different superscripts within a column indicate significantly different group mean amounts of PCV2 DNA (*P*<0.05).

In nasal swabs, PCV2 DNA was first detected in three of eight mPCV2 pigs, one of six PCV2b-234-K pigs and three of six reciprocal chimeric PCV2 pigs at 7 days p.i., and in the other groups by 14 days p.i. (Fig. 1b, Table 1). In general, the reciprocal chimeric PCV2 group shed the lowest amounts of PCV2 DNA by the nasal route with significant differences from PCV2a and PCV2b-234-K at 14 days p.i., and PCV2b at 21 days p.i. (Table 1).

In faecal swabs, PCV2 DNA was detected by 7 days p.i. in two of seven PCV2a pigs, three of seven PCV2b pigs, four of eight mPCV2 pigs, six of six PCV2b-234-K pigs and six of six reciprocal chimeric PCV2 pigs (Table 1). When comparing PCV2a, PCV2b and mPCV2 groups, significantly higher amounts of PCV2 DNA were excreted by mPCV2-infected pigs compared with PCV2b-infected pigs at 14 days p.i. (Fig. 1c). When considering all groups, the reciprocal chimeric PCV2b group had significantly lower levels compared with all groups except PCV2b at 7 days p.i. (Table 1).

### Gross lesions

Macroscopic lesions were characterized by mild-to-severe enlargement of lymph nodes in the majority of the pigs regardless of infection status. Several pigs (two of seven negative controls, three of seven PCV2a pigs, five of seven PCV2b pigs, two of six PCV2b-234-K pigs and one of six reciprocal chimeric PCV2 pigs) had lesions consistent with mild-to-severe chronic bacterial septicaemia (fibrinous peritonitis, pleuritis, and pericarditis, fibrin tags on the lung surface, and enlarged livers), and these pigs frequently also had craniovental consolidation and tan or purple discoloration of lung lobes resulting in lung scores of up to 43 % of the lung surface affected (data not shown). Mild-to-severe icterus, severe thymic atrophy, fatty liver and serous atrophy of fat were present in three of eight mPCV2 pigs, and severe, diffuse interlobular oedema was present in one of six reciprocal chimeric PCV2 pigs.

### Microscopic lesions, PCV2 antigen in tissues and PCVAD prevalence

Microscopic lesions were not present in lymphoid tissues of the negative controls and PCV2 antigen was also not detected in these pigs. The majority of the PCV2-infected pigs, regardless of PCV2 subtype, had severe lymphoid depletion of follicles and mild-to-moderate histiocytic replacement of follicles in multiple lymphoid tissues often associated with high amounts of PCV2 antigen (Table 2). An overall lymphoid lesions score of 7, 8 or 9, which is consistent with PCVAD (Opriessnig *et al.*, 2004), was evident in three of seven PCV2a pigs, four of seven PCV2b pigs, four of eight mPCV2 pigs, five of six PCV2b-234-K pigs and in one of six reciprocal chimeric PCV2 pigs. Moderate multifocal lymphohistiotyic hepatitis (score 2) was present in three of seven PCV2b pigs, one of eight mPCV2 pigs, one of six PCV2-234-K pigs and one of six reciprocal chimeric PCV2 pigs. Hepatic lesions were severe and diffuse (score 3) with moderate hepatocyte degeneration in three of eight mPCV2 pigs. Group mean hepatitis scores are summarized in Table 2.

**Table 2.** Mean±sem overall group PCVAD score, pigs in each PCVAD score category [*n* (%)], prevalence and group mean±sem log_10_PCV2 genomic copies in lymph nodes or lungs as determined by quantitative real-time (qRT)-PCR, prevalence and group mean±sem PCV2 antigen score (range: 0, negative; 3, abundant) as determined by immunohistochemistry (IHC), and prevalence and group mean±sem hepatitis score 21 days p.i.

Group designation	*n*	Mean overall group PCVAD score*	PCVAD score distribution				PCV2 DNA		PCV2 antigen in lymphoid tissues 0-3	Hepatitis 0-3
			Normal 0	Mild 1-3	Moderate 4-6	Severe 7-9	Lymph node	Lungs
Negative controls	7	0.0±0.0^A^	7 (100)	0 (0)	0 (0)	0 (0)	0/7 (0)^A^	0/7 (0)^A^	0/7 (0)^A^	3/7 (0.4±0.2)^A^
PCV2a	7	5.7±1.1^B,C^	0 (0)	2 (28.6)	2 (28.6)	3 (42.9)	7/7 (10.4±0.3)^B,C^	7/7 (9.4±0.5)^B^	7/7 (2.1±0.3)^B,C^	3/7 (0.4±0.2)^A^
PCV2b	7	7.1±0.6^B,C^	0 (0)	0 (0)	3 (42.8)	4 (57.1)	7/7 (10.6±0.3)^B^	7/7 (10.0±0.3)^B^	7/7 (2.5±0.2)^B^	6/7 (1.3±0.3)^B^
mPCV2	8	6.4±1.1^B.C^	0 (0)	2 (25)	2 (25)	4 (50)	8/8 (10.8±0.5)^B^	7/8 (9.0±1.4)^B^	8/8 (2.2±0.3)^B^	6/8 (1.6±0.5)^B^
PCV2b-234-K	6	8.5±0.5^B^	0 (0)	0 (0)	1 (16.7)	5 (83.3)	6/6 (10.6±0.2)^B^	7/7 (9.5±1.0)^B^	7/7 (2.8±0.2)^B^	6/6 (1.2±0.2)^B^
Reciprocal chimeric PCV2	6	3.8±1.3^C^	1 (16.7)	3 (50)	1 (16.7)	1 (16.7)	6/6 (9.0±.4)^C^	4/6 (5.5±1.8)^B^	5/6 (1.3±0.4)^C^	2/6 (0.5±0.3)^A,B^

Different superscripts within a column indicate significantly different group mean amounts of PCV2 DNA or antigen (*P*<0.05).

* A combined scoring system for each lymphoid tissue that ranged from 0 to 9 (lymphoid depletion score 0–3; inflammation score 0–3; PCV2 IHC score 0–3) was used to calculate the mean overall group PCVAD score. The combined score for all lymphoid tissues was divided by the total number of tissues.

### Prevalence and amount of PCV2 in lung and lymphoid tissues

PCV2 DNA was detectable in lymphoid tissues and the majority of the lung tissues in all infected pigs in all groups (Table 2). Similarly, PCV2 antigen was detected in lymphoid tissues in all but one infected pig (Table 2).

### Importance of the 234-K addition in ORF2

The log_10_ group mean PCV2 DNA genomic copies in serum samples at 21 days p.i. (8.4±0.3 versus 7.3±0.3), nasal swabs at 14 days p.i. (6.4±0.2 versus 5.7±0.2), and faecal swabs at 14 days p.i. (8.1±0.3 versus 6.3±0.5) and 21 days p.i. (6.3±0.4 versus 4.5±0.4) were significantly higher in ‘234-K’ virus-infected pigs compared with the ‘233’ virus-infected pigs. Moreover, PCVAD was diagnosed in 40 % (eight of 20) of the pigs infected with PCV2 strains that did not possess the 234-K addition in ORF2 and in 64.3 % (nine of 14) of the pigs that were infected with PCV2s containing a lysine at position 234 (*P* = 0.29).

## Discussion

Although the increasing use of molecular techniques and mathematical modelling has considerably advanced our knowledge on infection dynamics, the determination of differences in virus virulence still relies on utilization of *in vivo* models. In the present study, infectious DNA clones were constructed and used to produce biologically pure homogeneous infectious stocks of various PCV2 viruses in order to address and remove miscellaneous virus contaminations that may be present when utilizing virus stocks obtained from naturally infected pigs. In this study, we also used the CDCD pig model, which is known for its high sensitivity to virus pathogenicity. The main finding in the present study was the lack of marked differences in virulence between PCV2a, PCV2b and mPCV2 strains. All three viruses were capable of producing PCVAD in young CDCD pigs. Whilst this is in line with previous observations indicating that differences in virulence between PCV2a and PCV2b genotypes are lacking (Opriessnig *et al.*, 2008), it is in contrast to a Chinese study that suggested that mPCV2 is more virulent (Guo *et al.*, 2012). In that study, conventional pigs were utilized and infected with cell-culture-propagated PCV2 isolates recovered from field cases.

It is well recognized that highly virulent viruses are sometimes more difficult to propagate *in vitro.* In this regard for PCV2, PCV2b is often more difficult to grow in PK-15 cells compared with PCV2a (Guo *et al.*, 2011). Based on observations in our laboratory, the growth of mPCV2 in PK-15 cells is even further reduced. However, pigs infected experimentally with mPCV2 had earlier onset of viraemia and higher viral shedding compared with pigs infected with PCV2b. Similar to our study, the Chinese mPCV2-infected pigs also had an earlier onset of viraemia compared with PCV2a- and PCV2b-infected pigs; however, the majority of the pigs in the Chinese study developed detectable viraemia at ~14 days p.i., which is later than expected and later than we observed in the present study in pigs infected with infectious DNA clone-derived virus stocks. Differences in sensitivity of PCR assays, the source of the infectious virus stocks or infectious doses of the virus stocks utilized for inoculation between studies may be responsible for the discrepancies between studies. In the present study, a quantitative real-time (qRT)-PCR assay targeting a conserved region within ORF1 (Opriessnig *et al.*, 2003) was utilized for detection and quantification of the different PCV2s. The reverse primer and the probe matched 100 % at the nucleic acid level with the corresponding ORF1 region of the viruses in this study, whilst there was a nucleotide mismatch at location 15 of the forward primer (‘T’ in the primer versus ‘C’ in the PCV2 strains). This single mismatch affected all three strains utilized (PCV2a, PCV2b and mPCV2) and therefore the same efficiencies in amplification can be assumed (Süss *et al.*, 2009). Nevertheless, the increased ability of mPCV2 to replicate and shed early after infection was evident in both studies, and this may explain why mPCV2 appears to be on its way to becoming the predominant PCV2 genotype. In addition, by 21 days p.i. only the mPCV2-infected pigs had developed recognizable icterus, which was associated with severe microscopic hepatitis.

In this study, a young CDCD pig model was utilized and pigs were infected with infectious DNA clone-derived PCV2 isolates. Under field conditions, singular PCV2 infection is an exception rather than the rule (Opriessnig & Halbur, 2012) and the effect of co-infections of other swine pathogens on mPCV2 virulence needs to be further determined. Recently, no difference was found in viral growth and cytokine production when macrophages were infected with PCV2a and porcine reproductive and respiratory syndrome virus (PRRSV) or PCV2b and PRRSV using several type 1 and 2 PRRSV isolates (Sinha *et al.*, 2012). Similarly, no differences were detected when PCV2a- or PCV2b-infected pigs were concurrently infected with a type 2 PRRSV isolate (Sinha *et al.*, 2011). Emerging parvoviruses have been indicated as possible contributing factors to mPCV2-associated clinical manifestation of disease in vaccinated pigs (Opriessnig *et al.*, 2013), but this awaits further confirmation.

In this study, there were no significant differences in virulence among distinct classical and emerging PCV2 isolates using a CDCD pig model. All isolates utilized were capable of inducing severe disease in young pigs. Limitations of the study include the naive immune system of CDCD pigs and the young age at infection, which may have contributed to artificial acceleration of clinical disease in this model, and the short duration of the study, which may have prevented a more pronounced antibody response. Nevertheless, a higher frequency of PCVAD was observed in pigs infected with mPCV2 strains containing 234 aa in the capsid compared with those infected with PCV2 strains with 233 aa in this region, perhaps indicating some degree of difference, which was supported by significantly higher levels of PCV2 DNA in serum at 21 days p.i., nasal swabs at 14 days p.i., and faecal samples at 14 and 21 days p.i. in pigs that were infected with mPCV2 containing the 234-K addition in ORF2. Further investigations are warranted.

## Methods

### 

#### Generation of infectious virus DNA clones.

The construction of the infectious DNA clones for PCV2a (isolate 40895) and PCV2b (isolate NC16845) has been described previously (Beach *et al.*, 2010; Fenaux *et al.*, 2002). The full-length infectious DNA clone of the mPCV2 isolate JX535296 (Opriessnig *et al.*, 2013) was amplified by PCR using primers 1 and 3 (Table 3) from DNA extracted from a lung homogenate of a 2012 Iowa pig with severe PCVAD (Xiao *et al.*, 2012). The PCR product was blunt ligated to the pCR Blunt II TOPO vector using the Zero Blunt TOPO PCR Cloning kit (Life Technologies) as per the manufacturer’s instructions.

**Table 3.** Primers utilized for generation of the infectious virus clones

Primer ID	Primer sequence
1	5′-TTTCCGCGGGCTGGCTGAACTTTTGAAAG-3′
2	5′-ACCCCCCACTTAACCCTAAGTGAATAATAAAAACCATTAC-3′
3	5′-AGCCCGCGGAAATTTCTGACAAACGTTAC-3′
4	5′-CCTCCTTGGATACGTCATATCTGAAAACGAAAGAA-3′
5	5′-GTTTTTATTATTCACTTAGGGTTAAGTGGGGGG-3′
6	5′-CTTTCGTTTTCAGATATGACGTATCCAAGGAGGCG-3′
7	5′-CCACTTAACCCTTAATGAATAATAAAAACCATTAC-3′
8	5′-CCTCCTTGGATACGTCATCGCTGAAAACGAAAGAA-3′
9	5′-CTTTCGTTTTCAGCGATGACGTATCCAAGGAGGCG-3′
10	5′-GTTTTTATTATTCATTAAGGGTTAAGTGGGGGGT-3′
11	5′-TTTTTATCACTTCGTAATGGTTTTTATTATTCACTTAGGGTTAAGTGGGGGGT-3′
12	5′-ACCCCCCACTTAACCCTAAGTGAATAATAAAAACCATTACGAAGTGATAAAAA-3′

**Fig. 2.**
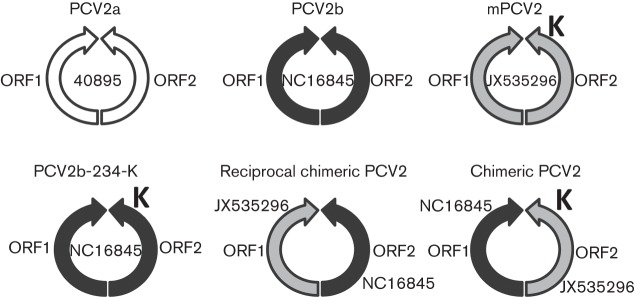
Schematic representation of the virus origins of the infectious clones constructed in this study. K indicates that there is an additional lysine in ORF2, which has a length of 234 aa residues instead of 233 aa.

In addition to PCV2a, PCV2b and mPCV2 infectious DNA clones, we also constructed several chimeric, reciprocal chimeric and mutant viruses between PCV2b and mPCV2 (Table 4, Fig. 2). To produce a classical PCV2b infectious DNA clone with a 234 aa ORF2 similar to that of mPCV2, the classical PCV2b strain NC16845 was modified by site-directed mutagenesis. In brief, the site-directed mutagenesis primers 11 and 12 were designed following the QuikChange mutagenesis kit guidelines (Agilent Technologies) and mutagenesis was performed per the QuikChange II Site-Directed Mutagenesis kit protocol (Agilent Technologies) on the NC16845-based PCV2b infectious DNA clone.

**Table 4.** Group designations, original viruses utilized and ORF origin of each virus construct

Group designation	No. of CDCD pigs	PCV2 strain	ORF1	ORF2
Negative controls	7	Saline	na	na
PCV2a	7	40895	PCV2a	PCV2a
PCV2b	7	NC16845	PCV2b	PCV2b
mPCV2	8	JX535296	mPCV2	mPCV2
PCV2b-234-K	6	NC16845 with lysine ORF2 position 234	PCV2b	PCV2b+K
Chimeric PCV2	6	NC16845 and JX535296	PCV2b	mPCV2
Reciprocal chimeric PCV2	6	JX535296 and NC16845	mPCV2	PCV2b

na, Not applicable.

To produce chimeric virus strains between PCV2b and mPCV2, the ORF1 and ORF2 of PCV2b strain NC16845 and mPCV2 strain JX535296 were utilized. In brief, overlapping extension PCRs were performed for NC16845 ORF1 and JX535296 ORF2 as well as JX535296 ORF1 and NC16845 ORF1 as described previously (Beach *et al.*, 2010). Full-length chimeric clones were produced by assembling three overlapping PCR fragments. For construction of chimeric PCV2b, the NC16845 template and primers 1 and 2 as well as primers 3 and 4 were utilized, and the JX535296 template and primers 5 and 6 were also used. For the construction of the reciprocal chimeric PCV2, the JX535296 template was used with primers 1 and 7 and primers 3 and 8, and the NC16845 template was used with primers 9 and 10. Each amplicon was produced using Accuzyme Mix (Bioline) (95 °C 10 min; 35 cycles of 95 °C 30 s, 54 °C 30 s, 68 °C 30 s, followed by 68 °C for 1.5 min). The fusion PCR was performed to assemble the full-length chimeric genomes (95 °C 10 min; 35 cycles of 95 °C 30 s, 60 °C 30 s, 68 °C 30 s, followed by 68 °C for 4 min). The full-length fusion products were blunt ligated to the pCR Blunt II TOPO vector using the Zero Blunt TOPO PCR Cloning kit (Life Technologies) per the manufacturer’s instructions. Each DNA clone was fully sequenced to confirm the authenticity of each construct.

#### Production of the infectious virus stocks.

A subclone of the PK-15 cell line free of PCV1 contamination as described previously (Fenaux *et al.*, 2002) was used to produce and titrate infectious virus stocks for this study. PCV2a, PCV2b, mPCV2, PCV2b-234-K, chimeric and reciprocal chimeric PCV2 virus stocks were obtained by isolation of each of the full-length virus genomes and concatemerization, followed by transfection of the concatemerized DNA clones into PK-15 cells as described previously (Opriessnig *et al.*, 2006b). Briefly, the full-length viral genomes were excised from the respective plasmid using the *Sac*II restriction enzyme. The digested genomes were self-ligated using the CloneDirect Rapid Ligation kit (Lucigen). The concatemerized viral genomes were then transfected into PK-15 cells and an immunofluorescence assay (IFA) with a PCV2-specific antibody was used to determine infectious virus titres as described previously (Fenaux *et al.*, 2002). Virus production was analysed by IFA using a mouse anti-PCV2 capsid primary mAb (Rural Technologies) and a goat anti-mouse FITC-labelled mAb (KPL).

#### Animals, housing and experimental design.

The experimental protocol was approved by the Iowa State University Institutional Animal Care and Use Committee. Forty-seven CDCD pigs were purchased at 2 weeks of age and arbitrarily assigned to one of seven groups and rooms with six to eight pigs in each group (Table 4). The pigs were housed in raised plastic decks equipped with one nipple drinker and one self-feeder. All groups were fed *ad libitum* with a balanced, pelleted feed ration (Nature’s Made). Virus challenge was done at 3 weeks of age. Each group was challenged with a different PCV2 strain as outlined in Table 4 by a combination of intramuscular and intranasal routes. At 6 weeks of age, corresponding to 21 days p.i., all pigs were humanely euthanized and a necropsy was conducted.

#### Clinical observation and mean daily weight gain.

All pigs were weighed at arrival at 2 weeks of age and at the time of necropsy at 6 weeks of age. The mean daily weight gain was calculated and compared among groups. All pigs were examined daily for signs of illness, including lethargy, respiratory signs, inappetence, icterus and lameness.

#### Virus challenge.

Following the determination of TCID_50_ titres for each virus using the Reed–Muench method, all virus stocks were adjusted to 10^3.66^ TCID_50_ ml^−1^ for the pig inoculations. The inoculum stock was each sequenced around the ORF2 region to verify the authenticity of respective virus stock. The inoculum stocks were stored at −80 °C until usage. Each pig received 5 ml of the respective inoculum (5×10^3.66^ TCID_50_ titre per pig) (Table 4) by a combination of the intranasal route (slowly dripping 1.5 ml into each of the two nostrils) and the intramuscular route (intramuscular injection of 2 ml into the right neck area).

#### Sample collection.

Blood was collected from all pigs prior to challenge, and again at 7, 14 and 21 days p.i. in 8.5 ml serum separator tubes (Fisher Scientific). The blood was centrifuged at 2000 ***g*** for 10 min at 4 °C and serum was stored at −80 °C until testing. In addition, nasal and faecal samples were collected weekly from each pig using polyester swabs (Fisher Scientific). Swabs were stored in 5 ml plastic tubes (Fisher Scientific) containing 1 ml sterile saline solution (Fisher Scientific). Lung tissues and inguinal lymph node samples were collected during necropsy at 21 days p.i., and stored in separate bags at −80 °C until testing.

#### Necropsy.

All pigs were humanely euthanized by intravenous pentobarbital sodium overdose (Fatal Plus; Vortech Pharmaceuticals) and necropsied at 21 days p.i. The extent of macroscopic lung lesions ranging from 0 to 100 % was scored as described previously (Halbur *et al.*, 1995). The sizes of superficial inguinal lymph nodes were compared among groups as described (Opriessnig *et al.*, 2006a). Sections of lymph nodes (superficial inguinal, external iliac, mediastinal, tracheobronchial and mesenteric), tonsil, heart, thymus, kidney, colon, spleen, liver, and small (ileum) and large (spiral colon) intestines were fixed in 10 % neutral-buffered formalin, and routinely processed for histological examination. In addition, lung and superficial inguinal lymph nodes were collected in separate bags, and stored at −80 °C for further PCR testing.

#### Serology.

Serum samples were tested by an ORF2-based PCV2 IgG ELISA as described previously (Nawagitgul *et al.*, 2002) and were considered positive if the sample-to-positive ratio was ≥0.2. Samples with sample-to-positive ratios between 0.1 and 0.2 were considered suspect.

#### DNA extraction.

Lung and superficial inguinal lymph node samples of ~1 g were minced and diluted 1 : 10 in Hank’s balanced salt solution, homogenized by using a Stomacher 80 (Seward Laboratory Systems) and centrifuged at 1500 ***g*** for 10 min to obtain supernatant. Total nucleic acids were extracted from serum samples, nasal swabs, faecal swabs, lung homogenates or superficial inguinal lymph node homogenates using the MagMax Pathogen RNA/DNA kit (Applied Biosystems) and an automated DNA/RNA extraction system (Kingfisher Flex; Thermo Fisher Scientific) according to the instructions of the manufacturer.

#### Detection and quantification of viral nucleic acids.

All serum samples, lung homogenates and superficial inguinal lymph node homogenates were tested for the presence of PCV2 DNA by qRT-PCR assays using primer/probe combinations as described (Opriessnig *et al.*, 2003; Shen *et al.*, 2010b) with the following modifications: a commercially available master mix (TaqMan Universal PCR Master Mix; Applied Biosystems) was used, the reaction volume was 25 µl, only one replica was tested for each sample, and the thermal cycler conditions were 50 °C for 2 min, 95 °C for 10 min, followed by 40 cycles of 95 °C for 10 s and 60 °C for 1 min. Samples were considered negative when no signal was observed within the 40 amplification cycles. Five serial dilutions of a PCV2 genomic DNA clone (10^5^–10^9^ copies ml^−1^) were used to generate a standard curve with a correlation coefficient of >0.99 (Opriessnig *et al.*, 2003).

#### Sequence confirmation of authentic inoculum.

All PCV2 DNA-positive serum samples were further tested with PCV2a/PCV2b/mPCV2 differential PCR assays (Opriessnig *et al.*, 2010, 2013) to determine the PCV2 genotype. The original inoculum stock and a PCV2 DNA-positive serum sample collected at 21 days p.i. in each group and room were sequenced. In brief, a conventional PCR covering the entire ORF2 was utilized as described previously (Gerber *et al.*, 2013). The obtained 768 bp products were sequenced at the Iowa State University DNA Facility. Sequences were aligned with published data using blast (http://www.ncbi.nlm.nih.gov/), and compiled using Lasergene 11 software and the clustal v alignment algorithm (DNASTAR).

#### Histopathology and immunohistochemistry (IHC).

Microscopic lesions were evaluated by two veterinary pathologists (T. O. and P. G. H.) blinded to the treatment group status. Lung sections were scored for the presence and severity of interstitial pneumonia, ranging from 0 (normal) to 6 (severe diffuse) (Halbur *et al.*, 1995). Sections of heart, liver, kidney, ileum, colon, brain and thymus were evaluated for the presence of inflammation, and scored from 0 (none) to 3 (severe). Lymph nodes, spleen and tonsil were evaluated for presence of lymphoid depletion and granulomatous replacement of follicles ranging from 0 (normal) to 3 (severe) (Opriessnig *et al.*, 2004). Detection of PCV2-specific antigen using IHC was performed on sections of lymph nodes, tonsil and spleen using a rabbit PCV2 polyclonal antiserum (Sorden *et al.*, 1999). PCV2 antigen scoring was done by a veterinary pathologist (T. O.) blinded to animal group designation. Scores ranged from 0 (no signal) to 3 (>50 % of lymphoid follicles contained cells with PCV2 antigen staining) (Opriessnig *et al.*, 2004).

#### Overall lymphoid lesion score.

The overall lymphoid lesion score was calculated as described previously (Opriessnig *et al.*, 2004). In brief, a combined scoring system for each lymphoid tissue that ranged from 0 to 9 (lymphoid depletion score 0–3; inflammation score 0–3; PCV2 IHC score 0–3) was used. The combined scores of the lymphoid tissues were divided by the number of tissues (Opriessnig *et al.*, 2004).

#### Statistical analysis.

For data analysis, JMP Pro software version 10.0.2 (SAS Institute) was used. Summary statistics were calculated for all the groups to assess the overall quality of the dataset, including normality. Statistical analysis of the data was performed by one-way ANOVA for continuous data (log_10_-transformed PCR data, ELISA data and mean daily weight gain). A *P* value <0.05 was set as the statistically significant level. A pairwise test using Tukey’s adjustment was subsequently performed to determine which differences among groups were statistically different. Serum qRT-PCR results (copies ml^−1^) were log_10_ transformed prior to statistical analysis. Non-repeated nominal data (histopathology scores) were assessed using a non-parametric Kruskal–Wallis one-way ANOVA; if there was a significant difference, pairwise Wilcoxon tests were used to evaluate differences among groups. Differences in prevalence were determined by using χ^2^ tests. In line with the objectives, PCV2a (*n* = 7), PCV2b (*n* = 8) and mPCV2 (*n* = 8) groups were compared initially. In a subsequent step, all groups were compared. For completeness, the importance of the 234 mutation in ORF2 was also evaluated by using the following groups: ORF2 233 (PCV2a, PCV2b and reciprocal chimeric PCV2 groups, *n* = 20) versus ORF2 234 (mPCV2 and PCV2b-234-K groups, *n* = 14).
